# Characterization of early host responses in adults with dengue disease

**DOI:** 10.1186/1471-2334-11-209

**Published:** 2011-08-02

**Authors:** Thomas Tolfvenstam, Anna Lindblom, Mark J Schreiber, Ling Ling, Angelia Chow, Eng Eong Ooi, Martin L Hibberd

**Affiliations:** 1 Genome Institute of Singapore, 60 Biopolis Street #02-01 Genome, Singapore, 138672, Singapore; 2 Infectious Disease Unit, Department of Medicine, Karolinska University Hospital, Karolinska Institutet, 17176, Stockholm, Sweden; 3 Novartis Institute for Tropical Diseases, 10 Biopolis Road #05-01 Chromos, Singapore, 138670, Singapore; 4 DUKE-NUS Graduate Medical School, 8 College Road, Singapore, 169857, Singapore

## Abstract

**Background:**

While dengue-elicited early and transient host responses preceding defervescence could shape the disease outcome and reveal mechanisms of the disease pathogenesis, assessment of these responses are difficult as patients rarely seek healthcare during the first days of benign fever and thus data are lacking.

**Methods:**

In this study, focusing on early recruitment, we performed whole-blood transcriptional profiling on denguevirus PCR positive patients sampled within 72 h of self-reported fever presentation (average 43 h, SD 18.6 h) and compared the signatures with autologous samples drawn at defervescence and convalescence and to control patients with fever of other etiology.

**Results:**

In the early dengue fever phase, a strong activation of the innate immune response related genes were seen that was absent at defervescence (4-7 days after fever debut), while at this second sampling genes related to biosynthesis and metabolism dominated. Transcripts relating to the adaptive immune response were over-expressed in the second sampling point with sustained activation at the third sampling. On an individual gene level, significant enrichment of transcripts early in dengue disease were chemokines CCL2 (MCP-1), CCL8 (MCP-2), CXCL10 (IP-10) and CCL3 (MIP-1α), antimicrobial peptide β-defensin 1 (DEFB1), desmosome/intermediate junction component plakoglobin (JUP) and a microRNA which may negatively regulate pro-inflammatory cytokines in dengue infected peripheral blood cells, mIR-147 (NMES1).

**Conclusions:**

These data show that the early response in patients mimics those previously described *in vitro*, where early assessment of transcriptional responses has been easily obtained. Several of the early transcripts identified may be affected by or mediate the pathogenesis and deserve further assessment at this timepoint in correlation to severe disease.

## Background

Dengue virus (DENV) is endemic throughout the tropics and in many subtropical parts of the world, causing significant human morbidity and mortality [1]. An understanding of the host response, as obtained from genome-wide transcriptional profiling of dengue infection may reveal unique patterns pertaining to specific disease outcomes and identify molecular mechanisms that could be targeted pharmacologically [2,3] and a number of studies of the host transcriptional responses to DENV infection have been performed. Comparing the results from *in-vitro *DENV-infected cells; HUVEC [4], non-small lung cancer cells [5], HepG2 [6,7]; primary human immune cells [8] and muscle satellite cells [9]; to whole blood or isolated mononuclear cells from DENV-infected patients [10-14], the first studies reports abundant transcripts related to innate immunity while the latter largely report non-immune related transcripts of ER-stress, oxidative metabolism and signal transduction. However, the human studies sampled patients at the time of hospital admission or after 4-5 days of illness, focusing on differences between clinical phenotypes. We hypothesized that earlier assessment following symptom presentation would be required to characterize the *in-vivo *dengue innate immune response and that the early host responses may reflect components of the disease pathogenesis.

## Methods

To characterize the early transcriptional response to DENV-infection *in-vivo*, we performed transcriptional profiling on 31 clinically undifferentiated DENV RT-PCR positive patients sampled within 72 h, 4-7 days and 3-4 weeks after self-reported fever onset, and compared the signatures with controls consisting of 26 matched febrile patients of other etiology (table 1). The time point of the 1st, 2nd and 3rd sampling was regarded as acute disease, defervescence and convalescence, respectively. All patients were identified in the early dengue infection and control study [15] in Singapore. The study was conducted in accordance with the declaration of Helsinki and approved by the NHG ethical review board (DSRB B/05/013). Individuals eligible for inclusion gave their written consent to participate in the study, were ≥18 years of age and presented ≤72 h from onset of fever ≥38°C. None of the DENV RT-PCR positive patients progressed to fulfill the criteria for severe dengue [16], while 20 of 31 (65%) were subsequently hospitalized after inclusion, the main admission criteria being thrombocytopenia [17] (table 2). Seven of these patients reported occurrence of mucosal bleeding. DENV-RNA was quantified by a Taqman-based PCR [18] after serum extraction (QIAamp Viral RNA mini kit, Qiagen). The mean viral copy number in the dengue DENV RT-PCR positive samples were 2.99 × 10^9 ^copies/mL (range 5.29 × 10^4^-3.15 × 10^10^). Viral serotype was determined by sequencing [19], the most common serotype being DENV3 (20 patients) followed by DENV1 (10 patients) and DENV2 (one patient). There was no finding of DENV4. DENV-specific IgG was detected by enzyme immunosorbent assay (EIA) (Panbio). Total whole-blood RNA (collected in pax-gene tubes, Becton Dickinson) was extracted (PAX-gene RNA kit, Qiagen) and amplified (Illumina^® ^TotalPrep™ RNA Amplification Kit, Ambion) before hybridization to a gene-expression array containing 23,961 RefSeq gene sequences (Illumina HumanRef-8 V1BeadChip, Illumina) [20]. Data was extracted (Bead Studio Software, Illumina) and normalized using one color array data (GenespringGX software, Silicon Genetics). Data transformation was corrected for low signal, with values recorded <0.01 increased to the minimum (0.01). Per-chip (mean) normalization accounted for chip variability by dividing all of the measurements on each chip by a 50^th ^percentile value. Per-gene normalization accounted for variability between probe sets for different genes. Genes that were not confidently detected in at least two samples were excluded, leaving 10,229 genes available for differential expression. Differentially expressed genes were selected from the normalized data using significance analysis of microarrays (SAM) [21] using a cutoff of 5%, with an additional filter of a minimum 2-fold difference in expression between groups, together with a minimum detection threshold for the gene in at least half of the patients in the smallest group. Pathway-analysis (IPA software, Ingenuity Systems) was performed to analyze enriched or less-abundant gene lists and the significance of the association was measured using right-tailed Fisher's exact test. The microarray data was deposited in NCBI's Gene Expression Omnibus [22] and accessible through GEO Series accession number [GSE28405]. Secreted mediators corresponding to hybridization-identified highly enriched transcripts (CCL2 (MCP-1), -8 (MCP-2), CXCL10 (IP-10) and CCL3 (MIP-1α)) were investigated for differential expression using Taqman assays (Applied Biosystems) [23] and the corresponding proteins quantitated in serum by Luminex bead-array (BioRad)(for CXCL10 also an EIA, R&D Systems) as per manufacturer's instructions. Taqman data were normalized for RNA loading levels by using 18 s quantitation as a reference and exported using SDS RQ Manager Software (Applied Biosystems). Relative (RQ) levels were exported and analyzed for significance using the Wilcoxon Rank Sum Test (assuming the data were non-normally distributed). Fold-change analysis was based on median levels.

**Table 1:** Characteristics of patients sampled for whole-blood RNA

	DENV RT-PCR positive	DENV RT-PCR negative
N	31	26
Duration of fever to 1^st ^sampling, hours	43 (13-72)	27 (3-72)
Age, years	43 (23-66)	43 (21-67)
Male, no. (%)	16 (52)	14 (54)
DENV IgG positive at inclusion, no. (%)	10/31 (32)	11/26 (42)
Duration of symptoms to recovery, days	11 (3-22)	6 (2-21)

Data are mean (range) values. DENV, dengue virus; the time point of the 2^nd ^sampling was regarded as defervescence. N/A, not applicable.

**Table 2:** Sequential hematological parameters of DENV RT-PCR positive patients

	1^st ^sampling	2^nd ^sampling	3^rd ^sampling
Time from last sampling, hours	N/A	79 (48-144)	363 (216-672)
White blood cell count, 10^9^/L	3.5 (1.2-8.8)	2.9 (1.3-6.5)	6.2 (2.4-9.8)
Hematocrit, (%)	42.4 (33.4-53.3)	43.7 (32.0-58.5)	40.3 (23.1-62.2)
Thrombocyte count, 10^9^/L	159 (17-309)	84 (8-237)	333 (88-625)

Data are mean (range) values, unless otherwise indicated.

## Results

**Figure 1:**
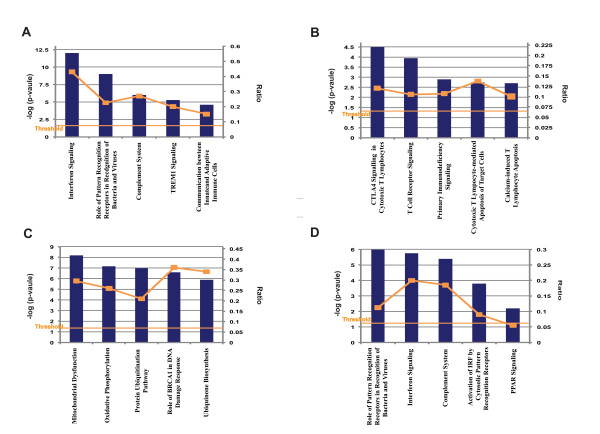
**Pathway analysis of differentially expressed transcripts in samples from patients with a) acute dengue as compared to patients with convalescent dengue (1,378 significantly enriched transcripts), b) acute dengue as compared to patients with convalescent dengue (575 significantly less enriched transcripts), c) dengue disease at defervescence as compared to patients with convalescent dengue disease (2,020 significantly enriched transcripts), d) acute dengue as compared to febrile patients with non-dengue disease (236 significantly enriched transcripts)**. Shown are the top five canonical pathways identified by unsupervised pathway analysis of filtered microarray data. The strength of the statistical association is indicated by the length of the bars. The ratio reflects the proportion of gene elements on the differentially expressed gene list that belong the specific canonical pathways.

By comparing samples drawn from DENV RT-PCR positive patients ≤72 h (on average sampled 43 h, SD 18.6 h, after self-reported fever debut), to autologous samples drawn 3-4 weeks after fever debut, we identified 1,378 significantly different abundant gene transcripts of which 803 were up-regulated and 575 down-regulated, relative to the convalescent sample (Additional file 1). Canonical pathway analyses of the significantly enriched genes revealed strongly significant up-regulated immune pathways as outlined in Figure 1a. The top two most significant canonical pathways were interferon (IFN)-signaling and pathways related to pattern recognition receptors. Other strongly over-represented innate immune responses were complement activation, TREM1 signaling, and communication between the innate and adaptive immune response. Among the down-regulated genes (n = 575) the top canonical pathways were T-cell associated pathways reflecting the relative up regulation of adaptive immunity during convalescence (Figure 1b). By comparing samples drawn from DENV RT-PCR positive patients 4-7 days (on average sampled 122 h, SD 30.8 h, after self-reported fever debut), to autologous samples drawn 3-4 weeks after fever debut, we could identify 2,677 differentially abundant genes of which 2,020 were up regulated and 657 down regulated (Additional file 2). Pathway analyses of the up regulated transcripts showed no immunity-related genes (Figure 1c). The dominating pathways relate to biosynthesis and metabolism functions. The statistical pathway association for the under-expressed genes was weak (data not shown). By comparing samples drawn from febrile DENV RT-PCR positive patients ≤72 h to samples drawn from febrile DENV RT-PCR negative patients ≤72 h (on average sampled 27 h, SD 24.0 h, after self-reported fever debut), we identified 536 significantly differently abundant gene transcripts of which 236 were enriched and 300 downregulated (Additional file 3). Canonical pathways analyses on the transcripts that were significantly enriched are outlined in Figure 1d. As in the analysis comparing patients with acute versus convalescent dengue disease, also here, the three most enriched canonical pathways that came up were pathogen recognition, IFN-signaling and complement activation. Pathway analysis of transcripts that were significantly less abundant found only canonical pathways with weak significant associations (data not shown). The most expressed transcripts and the associated pathways overlapped between the datasets, but fewer IFN-signaling and pathogen recognition associated transcripts were significantly expressed when comparing the datasets of acute dengue to non-dengue febrile illness. Several downstream genes in the type-1 IFN-signaling pathway were up-regulated in both datasets while type-2 IFN-signaling genes were overrepresented in the acute versus convalescent dengue disease comparison. Enrichment of toll-like receptor (TLR) 7 and MDA-5 transcripts was seen in both datasets. In the acute versus convalescent dengue disease comparison, the TLR6 receptor was over-expressed together with genes from the retinoic acid-inducible gene-I-like receptor (RLR) signaling pathway and the nod-like receptor (NLR) signaling pathway. Transcripts from genes clustering in the peroxisome proliderator-activated receptor (PPAR) signaling pathway were more significantly enriched when comparing acute dengue to non-dengue than when comparing acute versus convalescent dengue disaease. Among the transcripts representing secreted mediators, CCL2 was the most over-expressed transcript in both datasets, possibly due to an active feedback loop involving CCR1, JAK1 and STAT1/STAT3. In addition, transcripts of two other chemokines, CCL8 and CXCL10 and the antimicrobial peptide β-defensin 1 (DEFB1) were highly expressed in both datasets together with many components of the chemotactic network. Other highly expressed transcripts present in both datasets were; JUP (plakoglobin), NMES1 and CCNA1. Comparing whole genome expression profiles between patients with acute versus convalescent dengue disease indicated significant abundance of transcripts of the chemokines CCL2 (4,287 fold), CCL8 (160 fold), CXCL10 (30 fold) and CCL3 (17 fold) during acute dengue. All except MIP-1α were replicated, with smaller fold changes, in the comparison between febrile patients with acute dengue disease versus non-dengue disease. PCR quantification of the same transcripts in patients with acute versus convalescent dengue disease showed highly elevated fold changes for CCL2, -8 and CXCL10 but a more modest enrichment of MIP-1α at fold change 3.5 (table 3). Protein measurements of these chemokines in consecutive serum samples from the DENV RT-PCR positive patients showed significantly higher concentrations in acute dengue compared to convalescent dengue disease in correspondence with gene transcript fold changes (table 4). Comparing samples from DENV RT-PCR positive patients based on DENV-specific IgG status at inclusion (≤72 h after self-reported fever debut), one transcript was found significantly differently abundant, PRDX2 (accession NM_181738, 2-fold enriched in the ten DENV-IgG positive patients). Replicating this comparison of the autlogous samples collected at 4-7 days and 3-4 weeks after self-reported fever debut, four (all enriched in DENV-IgG positive patients) (Additional file 4) and 31 (18 enriched in DENV-IgG positive patients) (Additional file 5), were found significantly differently abundant, respectively. Pathway analysis of all these transcripts found only weakly significant canonical pathway associations (data not shown).

**Table 3:** Differential expression measured by taq-man low density array in DENV RT-PCR positive patients

	Fold change	
Gene	Acute dengue relative to convalescent dengue	Dengue at defervescence relative to convalescent dengue
CCL2	88.06	3.57
CCL8	169	3.48
CXCL10	44.26	6.64
CCL3	3.71	6.32

**Table 4:** Chemokine concentrations in consecutive serum samples from DENV RT-PCR positive patients

	Median concentration in serum (pg/mL)			
	Acute dengue disease	Dengue at defervescence	Convalescent dengue	*p*-value* acute vs convalescence
CCL2	268	104	62	<0.0001
CCL8	668	325	110	<0.0001
CXCL10	10750	7013	451	<0.0001
CCL3	8	nd	nd	

nd = under limit of detection, *Wilcoxon matched-pairs signed-ranks test.

## Discussion

Taken together, at this previously unassessed early stage of dengue disease the innate immune responses predominate, with the most significant canonical pathways being IFN-signaling, pattern recognition signaling and complement activation, both in relation to autologous transcripts in convalescence and to whole blood transcripts in non-dengue febrile illnesses at a similar phase following fever onset. In the convalescent phase of dengue disease, pathways related to adaptive immune responses are active, rendering these genes to appear down-regulated at the acute phase and in concordance with previous reports on transcripts derived from dengue disease in or just before defervescence [10-14], non-immune canonical pathways dominate. Interestingly, many of the responses seen in acute dengue in relation to the convalescent baseline were replicated when comparing to other non-dengue febrile illnesses, indicating a more prominent IFN-response, specific to dengue disease, and a selective utlization of TLR7, MDA5 and OAS. We do not have information of the etiology of the non-dengue febrile controls, but etiological search in patients enrolled later in the same cohort have shown that viral infections such as influenza, adenovirus and metapneumovirus are common among the DENV RT-PCR negative controls. IFN-responses arise hours after viremia is established and are likely of great importance for the control of viral replication. The IFN-response in dengue infection have been shown to be activated through two main pathogen recognition families; the TLRs and the RLRs. Of the TLRs, TLR7 in dendritic cells has been shown to interact with DENV RNA leading to viral fusion and uncoating processing that in the end activated a type-1 IFN-response [24]. TLR3 was also shown to have a role in the regulation of the inflammatory response in dengue infected umbilical vein endothelial cells [4]. When we compared the acute samples with the samples collected at convalescence in our dengue positive patients we saw an up regulation of TLR7 and IRF7 which indicates an activation of the TLR7 signalling pathway. TLR7 is an intracellular receptor that senses microbial nucleic acids and via IRF7 induces a strong type-1 IFN-response, particularly IFNα [25]; while it can also induced a type-1 IFN-response via NFκB [26]. RLR activation was also seen when comparing acute versus convalecent samples in the DENV RT-PCR positive patients, with the genes RIG1 and MDA5 upregulated. A recent study from Fredericksen et al demonstrated that RIG1 and MDA5 induce an IFN-response in West Nile virus-infected fibroblasts by activation of IRF3 [27]. They also show that RIG1 primes an early IFN-response while MDA5 is more involved in the second phase of IFN-dependent gene expression. Both RIG1 and MDA5 have also been shown to be activated in double RIG1/MDA5 knockout mouse fibroblasts [28,29]. Interestingly, RIPK2 (RIP2) was also identified as an over expressed gene indicating activation of the NLR signalling pathway. NOD1 and NOD2, members of the NLR protein family, are activated by specific bacterial peptides, and via RIPK2 induce NFκB activation [30]. There are no earlier reports on virus induced NLR activation. Transcripts clustering to the PPAR pathway were also among the most significantly enriched comparing dengue to non-dengue. PPARγ has been shown to play a critical role in the control of adipocyte differentiation and lipid metabolism, and also immunity and the barrier functions of epithelial and endothelial cells; in dengue disease this could be a response to epithelial stress [31,32]. Furthermore, among the most highly enriched gene transcripts is JUP (plakoglobin), that encode a protein that forms part of desmosomes and intermediate junctions in endothelial cells, wich could indicate that some transcripts were derived from affected endothelial cells. In fact, high numbers of detached endothelial cells in peripheral blood has been obseved in the acute phase of dengue [33]. DEFB1 was another highly enriched transcript which encodes an antimicrobial peptide, β- defensin 1, that has mostly been studied in the context of epithelial protection against HIV [34]. Also among the highly enriched transcripts were genes of the secreted mediators CCL2, CCL8, CXCL10 and CCL3, whose proteins were also found to be adundant in patient serum (Table 2). These have been observed before in dengue disease and MCP-1 could well participate in the pathogenesis of vascular leakage by its effect on endothelial tight junctions [35,36]. Other genes that were significantly enriched but did not cluster to any canonical pathway were NMES1 and CCNA1. While CCNA1 is a cyclin which probably mediates cell cycle arrest to prevent virus replication in infected cells, little is known about the gene NMES1. A recent study has identified that the transcript is a primary functional microRNA (miR-147) which was expressed in murine macrophages upon TLR-stimulation [37]. It was shown to negatively regulate inflammatory cytokine expression in these cells. As dengue virus is believed to primarily replicate in cells of monocyte/macrophage lineage, NMES1 expression may be important to moderate the pro-inflammatory cytokine release from these cells, especially as these are potential mediators of pathology. Comparing dengue patients with different DENV-serostatus at inclusion rendered a surprisingly small number of differentially expressed genes with no significant pathway clustering. On the individual gene level PRDX2, an antioxidant enzyme was enriched in the acute stage of secondary DENV infection, while at defervesence, CCR2 was among the transcripts seen upregulated in secondary infection. Sierra et al. addressed this issue by examining in-vitro infected PBMC from immune and non-immune individuals and showed expression of CCL2 to be highly dependent on previously infecting serotype at 24 h post infection [38]. CCL2 was not an found an enriched transcript in acute secondary infection in our material but the number of patients in each group in this specific comparison was small (ten patients were DENV RT-PCR and DENV-IgG positive) and information of previous infecting serotype was lacking, thus this specific assessment should be perfomed in a larger cohort to allow further speculation on these genes in the context of dengue pathogenesis.

## Conclusions

In summary, early DENV-induced transcriptional host responses *in-vivo *are predominatly involving innate immune responses and overlaps to a large extent with those described in *in-vitro*, where early transcriptional assessment post-infection has been easily obtained. Several of the early transcripts identified deserve further assessment in correlation to severe disease.

## Competing interests

The author declares that they have no competing interests.

## Authors' contributions

TT, EEO and MLH conceived and designed the study. LL and AC performed the laboratory analysis. TT, AL and MJS analyzed the data and drafted the manuscript. All authors participated in revising the manuscript. All authors have read and approved the final manuscript.

## Pre-publication history

The pre-publication history for this paper can be accessed here:

http://www.biomedcentral.com/1471-2334/11/209/prepub

## Supplementary Material

Additional file 1 **The top 100 differentially abundant transcripts in samples from patients with acute dengue relative to samples from patients with convalescent dengue**. A table outlining the top 100 differentially abundant transcripts in samples from patients with acute dengue relative to samples from patients with convalescent dengue. Click here for file

Additional file 2 **The top 100 differentially abundant transcripts in samples from patients with dengue disease at defervescence relative to samples from patients with convalescent dengue**. A table outlining the top 100 differentially abundant transcripts in samples from patients with dengue disease at defervescence relative to samples from patients with convalescent dengue. Click here for file

Additional file 3 **The top 100 differentially abundant transcripts in samples from patients with acute dengue relative to samples from febrile patients with non-dengue disease**. A table outlining the top 100 differentially abundant transcripts in samples from patients with acute dengue relative to samples from febrile patients with non-dengue disease. Click here for file

Additional file 4 **Differentially abundant transcripts in samples taken at defervescence from DENV RT-PCR/DENV-IgG positive patients at inclusion relative to DENV RT-PCR positive/DENV-IgG negative patients at inclusion**. A table outlining the differentially abundant transcripts in samples taken at defervescence from DENV RT-PCR/DENV-IgG positive patients at inclusion relative to DENV RT-PCR positive/DENV-IgG negative patients at inclusion. Click here for file

Additional file 5 **Differentially abundant transcripts in samples taken at convalescence from DENV RT-PCR/DENV-IgG positive patients at inclusion relative to DENV RT-PCR positive/DENV-IgG negative patients at inclusion**. A table outlining the differentially abundant transcripts in samples taken at convalescence from DENV RT-PCR/DENV-IgG positive patients at inclusion relative to DENV RT-PCR positive/DENV-IgG negative patients at inclusion. Click here for file

## References

[B1] Global burden of dengue http://www.pdvi.org

[B2] Jenner RG Young RA Insights into host responses against pathogens from transcriptional profiling Nat Rev Microbiol 2005 3 281 294 10.1038/nrmicro1126 15806094

[B3] Katze MG Fornek JL Palermo RE Walters KA Korth MJ Innate immune modulation by RNA viruses: emerging insights from functional genomics Nat Rev Immunol 2008 8 644 654 10.1038/nri2377 18654572 PMC7097543

[B4] Warke RV Xhaja K Martin KJ Fournier MF Shaw SK Brizuela N de Bosch N Lapointe D Ennis FA Rothman AL Bosch I Dengue virus induces novel changes in gene expression of human umbilical vein endothelial cells J Virol 2003 77 11822 11832 10.1128/JVI.77.21.11822-11832.2003 14557666 PMC229255

[B5] Nasirudeen AM Liu DX Gene expression profiling by microarray analysis reveals an important role for caspase-1 in dengue virus-induced p53-mediated apoptosis J Med Virol 2009 81 1069 1081 10.1002/jmv.21486 19382257

[B6] Conceicao TM El-Bacha T Villas-Boas CS Coello G Ramirez J Montero-Lomeli M Da Poian AT Gene expression analysis during dengue virus infection in HepG2 cells reveals virus control of innate immune response J Infect 2009 60 65 75 19837110 10.1016/j.jinf.2009.10.003

[B7] Fink J Gu F Ling L Tolfvenstam T Olfat F Chin KC Aw P George J Kuznetsov VA Schreiber M Vasudevan SG Hibberd ML Host gene expression profiling of dengue virus infection in cell lines and patients PLoS Negl Trop Dis 2007 1 e86 10.1371/journal.pntd.0000086 18060089 PMC2100376

[B8] Becerra A Warke RV Martin K Xhaja K de Bosch N Rothman AL Bosch I Gene expression profiling of dengue infected human primary cells identifies secreted mediators in vivo J Med Virol 2009 81 1403 1411 10.1002/jmv.21538 19551822 PMC2893327

[B9] Warke RV Becerra A Zawadzka A Schmidt DJ Martin KJ Giaya K Dinsmore JH Woda M Hendricks G Levine T Rothman AL Bosch I Efficient dengue virus (DENV) infection of human muscle satellite cells upregulates type I interferon response genes and differentially modulates MHC I expression on bystander and DENV-infected cells J Gen Virol 2008 89 1605 1615 10.1099/vir.0.2008/000968-0 18559930

[B10] Long HT Hibberd ML Hien TT Dung NM Van Ngoc T Farrar J Wills B Simmons CP Patterns of gene transcript abundance in the blood of children with severe or uncomplicated dengue highlight differences in disease evolution and host response to dengue virus infection J Infect Dis 2009 199 537 546 10.1086/596507 19138155 PMC4333209

[B11] Nascimento EJ Braga-Neto U Calzavara-Silva CE Gomes AL Abath FG Brito CA Cordeiro MT Silva AM Magalhaes C Andrade R Gil LH Marques ET Jr Gene expression profiling during early acute febrile stage of dengue infection can predict the disease outcome PLoS One 2009 4 e7892 10.1371/journal.pone.0007892 19936257 PMC2775946

[B12] Simmons CP Popper S Dolocek C Chau TN Griffiths M Dung NT Long TH Hoang DM Chau NV Thao le TT Hien TT Relman DA Farrar J Patterns of host genome-wide gene transcript abundance in the peripheral blood of patients with acute dengue hemorrhagic fever J Infect Dis 2007 195 1097 1107 10.1086/512162 17357045 PMC4042601

[B13] Ubol S Masrinoul P Chaijaruwanich J Kalayanarooj S Charoensirisuthikul T Kasisith J Differences in global gene expression in peripheral blood mononuclear cells indicate a significant role of the innate responses in progression of dengue fever but not dengue hemorrhagic fever J Infect Dis 2008 197 1459 1467 10.1086/587699 18444802

[B14] Loke P Hammond SN Leung JM Kim CC Batra S Rocha C Balmaseda A Harris E Gene expression patterns of dengue virus-infected children from nicaragua reveal a distinct signature of increased metabolism PLoS Negl Trop Dis 2010 4 e710 10.1371/journal.pntd.0000710 20559541 PMC2886038

[B15] Low JG Ooi EE Tolfvenstam T Leo YS Hibberd ML Ng LC Lai YL Yap GS Li CS Vasudevan SG Ong A Early Dengue infection and outcome study (EDEN) - study design and preliminary findings Ann Acad Med Singapore 2006 35 783 789 17160194

[B16] Dengue: guidelines for diagnosis, treatment, prevention and control -- New edition http://whqlibdoc.who.int/publications/2009/9789241547871_eng.pdf 23762963

[B17] Lye DC Chan M Lee VJ Leo YS Do young adults with uncomplicated dengue fever need hospitalisation? A retrospective analysis of clinical and laboratory features Singapore Med J 2008 49 476 479 18581021

[B18] Ito M Takasaki T Yamada K Nerome R Tajima S Kurane I Development and evaluation of fluorogenic TaqMan reverse transcriptase PCR assays for detection of dengue virus types 1 to 4 J Clin Microbiol 2004 42 5935 5937 10.1128/JCM.42.12.5935-5937.2004 15583346 PMC535301

[B19] Schreiber MJ Holmes EC Ong SH Soh HS Liu W Tanner L Aw PP Tan HC Ng LC Leo YS Low JG Ong A Ooi EE Vasudevan SG Hibberd ML Genomic epidemiology of a dengue virus epidemic in urban Singapore J Virol 2009 83 4163 4173 10.1128/JVI.02445-08 19211734 PMC2668455

[B20] Hoang LT Lynn DJ Henn M Birren BW Lennon NJ Le PT Duong KT Nguyen TT Mai LN Farrar JJ Hibberd ML Simmons CP The early whole-blood transcriptional signature of dengue virus and features associated with progression to dengue shock syndrome in Vietnamese children and young adults J Virol 2010 84 12982 12994 10.1128/JVI.01224-10 20943967 PMC3004338

[B21] Tusher VG Tibshirani R Chu G Significance analysis of microarrays applied to the ionizing radiation response Proc Natl Acad Sci USA 2001 98 5116 5121 10.1073/pnas.091062498 11309499 PMC33173

[B22] Edgar R Domrachev M Lash AE Gene Expression Omnibus: NCBI gene expression and hybridization array data repository Nucleic Acids Res 2002 30 207 210 10.1093/nar/30.1.207 11752295 PMC99122

[B23] Burgner D Davila S Breunis WB Ng SB Li Y Bonnard C Ling L Wright VJ Thalamuthu A Odam M Shimizu C Burns JC Levin M Kuijpers TW Hibberd ML A genome-wide association study identifies novel and functionally related susceptibility Loci for Kawasaki disease PLoS Genet 2009 5 e1000319 10.1371/journal.pgen.1000319 19132087 PMC2607021

[B24] Wang JP Liu P Latz E Golenbock DT Finberg RW Libraty DH Flavivirus activation of plasmacytoid dendritic cells delineates key elements of TLR7 signaling beyond endosomal recognition J Immunol 2006 177 7114 7121 17082628 10.4049/jimmunol.177.10.7114

[B25] Kumar H Kawai T Akira S Toll-like receptors and innate immunity Biochem Biophys Res Commun 2009 388 621 625 10.1016/j.bbrc.2009.08.062 19686699

[B26] Diamond MS Mechanisms of evasion of the type I interferon antiviral response by flaviviruses J Interferon Cytokine Res 2009 29 521 530 10.1089/jir.2009.0069 19694536

[B27] Fredericksen BL Keller BC Fornek J Katze MG Gale M Jr Establishment and maintenance of the innate antiviral response to West Nile Virus involves both RIG-I and MDA5 signaling through IPS-1 J Virol 2008 82 609 616 10.1128/JVI.01305-07 17977974 PMC2224571

[B28] Chang TH Liao CL Lin YL Flavivirus induces interferon-beta gene expression through a pathway involving RIG-I-dependent IRF-3 and PI3K-dependent NF-kappaB activation Microbes Infect 2006 8 157 171 10.1016/j.micinf.2005.06.014 16182584

[B29] Loo YM Fornek J Crochet N Bajwa G Perwitasari O Martinez-Sobrido L Akira S Gill MA Garcia-Sastre A Katze MG Gale M Jr Distinct RIG-I and MDA5 signaling by RNA viruses in innate immunity J Virol 2008 82 335 345 10.1128/JVI.01080-07 17942531 PMC2224404

[B30] Krieg A Correa RG Garrison JB Le Negrate G Welsh K Huang Z Knoefel WT Reed JC XIAP mediates NOD signaling via interaction with RIP2 Proc Natl Acad Sci USA 2009 106 14524 14529 10.1073/pnas.0907131106 19667203 PMC2732880

[B31] Huang W Eum SY Andras IE Hennig B Toborek M PPARalpha and PPARgamma attenuate HIV-induced dysregulation of tight junction proteins by modulations of matrix metalloproteinase and proteasome activities FASEB J 2009 23 1596 1606 10.1096/fj.08-121624 19141539 PMC2669424

[B32] Ogasawara N Kojima T Go M Ohkuni T Koizumi J Kamekura R Masaki T Murata M Tanaka S Fuchimoto J Himi T Sawada N PPARgamma agonists upregulate the barrier function of tight junctions via a PKC pathway in human nasal epithelial cells Pharmacol Res 2010 61 489 498 10.1016/j.phrs.2010.03.002 20227502

[B33] Cardier JE Rivas B Romano E Rothman AL Perez-Perez C Ochoa M Caceres AM Cardier M Guevara N Giovannetti R Evidence of vascular damage in dengue disease: demonstration of high levels of soluble cell adhesion molecules and circulating endothelial cells Endothelium 2006 13 335 340 10.1080/10623320600972135 17090406

[B34] Prado-Montes de Oca E Human beta-defensin 1: a restless warrior against allergies, infections and cancer Int J Biochem Cell Biol 2010 42 800 804 10.1016/j.biocel.2010.01.021 20100591

[B35] Lee YR Liu MT Lei HY Liu CC Wu JM Tung YC Lin YS Yeh TM Chen SH Liu HS MCP-1, a highly expressed chemokine in dengue haemorrhagic fever/dengue shock syndrome patients, may cause permeability change, possibly through reduced tight junctions of vascular endothelium cells J Gen Virol 2006 87 3623 3630 10.1099/vir.0.82093-0 17098977

[B36] Stamatovic SM Keep RF Kunkel SL Andjelkovic AV Potential role of MCP-1 in endothelial cell tight junction 'opening': signaling via Rho and Rho kinase J Cell Sci 2003 116 4615 4628 10.1242/jcs.00755 14576355

[B37] Liu G Friggeri A Yang Y Park YJ Tsuruta Y Abraham E miR-147, a microRNA that is induced upon Toll-like receptor stimulation, regulates murine macrophage inflammatory responses Proc Natl Acad Sci USA 2009 106 15819 15824 10.1073/pnas.0901216106 19721002 PMC2747202

[B38] Sierra B Perez AB Vogt K Garcia G Schmolke K Aguirre E Alvarez M Volk HD Guzman MG MCP-1 and MIP-1alpha expression in a model resembling early immune response to dengue Cytokine 2010 52 175 183 10.1016/j.cyto.2010.06.010 20650649

