# Supplementary material for: Characterization of early host responses in adults with dengue disease
Source: BMC Infect Dis. 2011 Aug 2;11:209. doi: 10.1186/1471-2334-11-209 (PMC3163546; doi:10.1186/1471-2334-11-209)
Supplement: Additional file 1 — The top 100 differentially abundant transcripts in samples from patients with acute dengue relative to samples from patients with convalescent dengue. A table outlining the top 100 differentially abundant transcripts in samples from patients with acute dengue relative to samples from patients with convalescent dengue. [file 1471-2334-11-209-S1.DOC]

Additional file 1. The top 100 differentially abundant transcripts in samples from patients with acute dengue relative to samples from patients with convalescent dengue.

| **Symbol** | **Fold change** | **NCBI accession** |  | **Symbol** | **Fold change** | **NCBI accession** |
| --- | --- | --- | --- | --- | --- | --- |
| CCL2 | 4,287.9 | NM_002982 |  | FLJ20701 | -63.5 | NM_017933 |
| NMES1 | 381.2 | NM_032413 |  | CD8B1 | -19.7 | NM_172100 |
| DEFB1 | 229.3 | NM_005218 |  | KIAA0450 | -16.6 | XM_371214 |
| LOC400759 | 167.8 | XM_375746 |  | PI3 | -15.1 | NM_002638 |
| MT1K | 165.2 | NM_176870 |  | PTGDR | -14.4 | NM_000953 |
| CCL8 | 160.0 | NM_005623 |  | ANKH | -13.0 | NM_054027 |
| CCNA1 | 156.6 | NM_003914 |  | NELL2 | -12.9 | NM_006159 |
| JUP | 149.5 | NM_002230 |  | C9orf45 | -11.9 | NM_030814 |
| BF | 146.7 | NM_001710 |  | CYP27A1 | -10.6 | NM_000784 |
| HINT3 | 120.6 | NM_138571 |  | SLC4A7 | -10.6 | NM_003615 |
| TGM1 | 98.0 | NM_000359 |  | C20orf100 | -10.2 | NM_032883 |
| LAMP3 | 87.6 | NM_014398 |  | LOC374565 | -9.9 | XM_353072 |
| PKD2L1 | 82.4 | NM_016112 |  | CRIP2 | -8.9 | NM_001312 |
| ABTB2 | 77.7 | NM_145804 |  | CD1C | -8.9 | NM_001765 |
| LOC375110 | 76.8 | XM_353248 |  | VENTX2 | -8.8 | NM_014468 |
| ZNF366 | 73.0 | NM_152625 |  | FLJ12592 | -8.5 | NM_032169 |
| LOC377812 | 72.5 | XM_352798 |  | LOC93622 | -8.4 | NM_138699 |
| FOXC1 | 43.9 | NM_001453 |  | KRTAP11-1 | -8.0 | NM_175858 |
| SIGLEC11 | 41.3 | NM_052884 |  | TNFSF5 | -7.9 | NM_000074 |
| TRIM14 | 40.7 | NM_014788 |  | ORM1 | -7.2 | NM_000607 |
| FLJ11577 | 38.5 | NM_025159 |  | BLR1 | -7.2 | NM_032966 |
| TRIM6 | 35.3 | NM_058166 |  | SPAP1 | -7.2 | NM_138739 |
| PML | 35.2 | NM_033247 |  | LOC283663 | -7.0 | XM_378514 |
| KIF23 | 32.7 | NM_004856 |  | GAMT | -6.9 | NM_138924 |
| FCGR1A | 32.0 | NM_000566 |  | BDH | -6.2 | NM_203315 |
| USP18 | 31.9 | NM_017414 |  | NMT2 | -5.9 | NM_004808 |
| G0S2 | 31.0 | NM_015714 |  | LOC388077 | -5.8 | XM_370834 |
| JAK2 | 30.9 | NM_004972 |  | VPREB3 | -5.8 | NM_013378 |
| CXCL10 | 30.1 | NM_001565 |  | LDOC1 | -5.7 | NM_012317 |
| IMP-3 | 28.1 | NM_006547 |  | MARLIN1 | -5.7 | NM_144720 |
| PLA2G4C | 26.2 | NM_003706 |  | WBSCR20C | -5.6 | NM_148980 |
| PI4K2B | 24.1 | NM_018323 |  | LOC349136 | -5.5 | NM_198285 |
| CPEB3 | 24.1 | NM_014912 |  | FLJ20898 | -5.5 | NM_024600 |
| STI2 | 23.6 | NM_145755 |  | NOV | -5.5 | X96584 |
| SAMD4 | 23.6 | NM_015589 |  | GAMT | -5.5 | NM_138924 |
| RGL1 | 23.6 | NM_015149 |  | KLRB1 | -5.5 | NM_002258 |
| PNUTL2 | 22.6 | NM_080417 |  | SELENBP1 | -5.4 | NM_003944 |
| TCN2 | 21.9 | NM_000355 |  | CDR2 | -5.2 | XM_071866 |
| C1QG | 21.2 | NM_172369 |  | NAV1 | -5.2 | NM_020443 |
| KIAA1618 | 21.1 | NM_020954 |  | LTBP3 | -5.1 | NM_021070 |
| HESX1 | 20.8 | NM_003865 |  | SLC4A3 | -5.1 | NM_005070 |
| CTSL | 20.0 | NM_001912 |  | C20orf36 | -5.1 | NM_018257 |
| DNAPTP6 | 19.5 | NM_015535 |  | INADL | -5.0 | NM_170605 |
| C6orf206 | 18.4 | NM_152732 |  | MGC29671 | -5.0 | NM_182538 |
| Hes4 | 17.5 | NM_021170 |  | MDS2 | -4.9 | NM_148895 |
| MDK | 17.3 | NM_002391 |  | LOC129293 | -4.8 | XM_059341 |
| CCL3 | 17.2 | NM_002983 |  | MGC33414 | -4.7 | NM_173574 |
| INDO | 16.9 | NM_002164 |  | UGT2A1 | -4.7 | NM_006798 |
| TRIM5 | 16.8 | NM_033092 |  | LOC378204 | -4.6 | XM_353706 |
| MGC12909 | 16.1 | NM_145055 |  | XCL1 | -4.6 | NM_002995 |
| GPR84 | 15.4 | NM_020370 |  | LCHN | -4.6 | XM_379938 |
| MT1J | 15.3 | NM_175622 |  | LOC222901 | -4.6 | XM_167275 |
| FLJ25084 | 14.6 | NM_152792 |  | FLJ40584 | -4.5 | XM_069189 |
| AKAP2 | 14.1 | NM_007203 |  | HSF2 | -4.5 | NM_004506 |
| OTOF | 14.0 | NM_194322 |  | SLC6A8 | -4.5 | NM_005629 |
| IL4I1 | 13.6 | NM_172374 |  | CD8A | -4.4 | NM_001768 |
| C9orf91 | 13.5 | NM_153045 |  | APBB1 | -4.4 | NM_145689 |
| RAB8B | 13.5 | NM_016530 |  | GRCC10 | -4.4 | NM_138425 |
| LOC388274 | 13.5 | XM_373684 |  | DPEP3 | -4.2 | NM_022357 |
| SERPING1 | 13.0 | NM_000062 |  | CA4 | -4.2 | NM_000717 |
| C12orf6 | 12.3 | NM_020367 |  | MME | -4.2 | NM_000902 |
| GBP1 | 11.9 | NM_002053 |  | CXCR3 | -4.2 | NM_001504 |
| CCRL2 | 11.8 | NM_003965 |  | ORC2L | -4.2 | NM_006190 |
| LOC401485 | 11.6 | XM_376809 |  | DTX3 | -4.2 | NM_178502 |
| MT2A | 11.4 | NM_005953 |  | KLC2L | -4.2 | NM_145275 |
| C1QB | 11.3 | NM_000491 |  | FLJ11383 | -4.1 | NM_024938 |
| PLSCR1 | 11.1 | NM_021105 |  | PLEKHB1 | -4.1 | NM_021200 |
| FRMD3 | 11.1 | NM_174938 |  | GPR44 | -4.0 | NM_004778 |
| LOC285510 | 10.9 | XM_209643 |  | ELYS | -4.0 | NM_175865 |
| CASP7 | 10.7 | NM_033340 |  | KLRG1 | -4.0 | NM_005810 |
| SOCS1 | 10.7 | NM_003745 |  | KIAA0114 | -4.0 | XM_353348 |
| TOR1B | 10.6 | NM_014506 |  | FLJ10359 | -3.9 | XM_375853 |
| RIN2 | 10.3 | NM_018993 |  | FCER1A | -3.9 | NM_002001 |
| AXL | 10.3 | M76125 |  | MS4A1 | -3.9 | NM_021950 |
| IL1RN | 9.9 | NM_173842 |  | MGC10870 | -3.9 | NM_032301 |
| LOC129607 | 9.8 | XM_059368 |  | GYLTL1B | -3.8 | NM_152312 |
| P2RY6 | 9.6 | NM_176798 |  | PASK | -3.8 | NM_015148 |
| NEXN | 9.5 | NM_144573 |  | ITLN1 | -3.8 | NM_017625 |
| IFI27 | 9.4 | NM_005532 |  | RPL23 | -3.8 | NM_000978 |
| PLAUR | 9.4 | NM_002659 |  | BCL2L1 | -3.8 | NM_138578 |
| XRN1 | 9.3 | NM_019001 |  | TLP19 | -3.8 | NM_015913 |
| ABCD1 | 9.2 | NM_000033 |  | ARHGEF9 | -3.8 | NM_015185 |
| GM2A | 9.1 | NM_000405 |  | ALS2CR2 | -3.7 | NM_018571 |
| PKD1-like | 9.0 | NM_182686 |  | EPHA1 | -3.7 | M18391 |
| cig5 | 8.9 | NM_080657 |  | EBI2 | -3.7 | NM_004951 |
| MTHFD2 | 8.9 | NM_006636 |  | CTLA4 | -3.7 | NM_005214 |
| IFRG28 | 8.9 | NM_022147 |  | MGC18216 | -3.7 | NM_152452 |
| HIST1H2AD | 8.9 | NM_021065 |  | BRI3BP | -3.6 | NM_080626 |
| TUFT1 | 8.8 | NM_020127 |  | MUC20 | -3.6 | NM_152673 |
| KIAA1463 | 8.7 | NM_020849 |  | SPAP1 | -3.6 | NM_138739 |
| LGALS3BP | 8.6 | NM_005567 |  | ZNF302 | -3.6 | NM_018675 |
| ANKFY1 | 8.5 | NM_020740 |  | C18orf1 | -3.6 | NM_004338 |
| GP1BA | 8.5 | NM_000173 |  | HSD17B8 | -3.6 | NM_014234 |
| NCOA7 | 8.4 | NM_181782 |  | SLC7A6 | -3.6 | NM_003983 |
| MEIS1 | 8.4 | NM_002398 |  | KIAA0794 | -3.6 | XM_087353 |
| KIF1B | 8.4 | NM_015074 |  | RPS29 | -3.6 | NM_001032 |
| G1P2 | 8.3 | NM_005101 |  | SLC4A1 | -3.5 | NM_000342 |
| TIMM10 | 8.1 | NM_012456 |  | RPLP0 | -3.5 | NM_001002 |
| SLC25A13 | 8.1 | NM_014251 |  | SESN3 | -3.5 | NM_144665 |
| TNNT1 | 8.1 | NM_003283 |  | CAMK1D | -3.5 | NM_153498 |
