# Supplementary material for: Characterization of early host responses in adults with dengue disease
Source: BMC Infect Dis. 2011 Aug 2;11:209. doi: 10.1186/1471-2334-11-209 (PMC3163546; doi:10.1186/1471-2334-11-209)
Supplement: Additional file 2 — The top 100 differentially abundant transcripts in samples from patients with dengue disease at defervescence relative to samples from patients with convalescent dengue. A table outlining the top 100 differentially abundant transcripts in samples from patients with dengue disease at defervescence relative to samples from patients with convalescent dengue. [file 1471-2334-11-209-S2.DOC]

Additional file 2. The top 100 differentially abundant transcripts in samples from patients with dengue disease at defervescence relative to samples from patients with convalescent dengue.

| **Symbol** | **Fold change** | **NCBI accession** |  | **Symbol** | **Fold change** | **NCBI accession** |
| --- | --- | --- | --- | --- | --- | --- |
| MGC3130 | 483.7 | NM_024032 |  | SLC4A3 | -32.7 | NM_005070 |
| BRRN1 | 456.6 | NM_015341 |  | SELENBP1 | -25.7 | NM_003944 |
| CDCA5 | 369.5 | NM_080668 |  | SLC4A1 | -20.8 | NM_000342 |
| KIAA0101 | 304.3 | NM_014736 |  | DKFZP434H | -18.8 | XM_290829 |
| BUB1 | 289.8 | NM_004336 |  | SESN3 | -18.6 | NM_144665 |
| RNASE1 | 261.0 | NM_198232 |  | SNCA | -17.2 | NM_007308 |
| PTTG3 | 248.2 | NM_021000 |  | PI3 | -15.3 | NM_002638 |
| PLK4 | 225.5 | NM_014264 |  | EPB42 | -14.8 | NM_000119 |
| DKFZp762E1312 | 223.1 | NM_018410 |  | IL18BP | -14.1 | NM_173044 |
| TPX2 | 210.8 | NM_012112 |  | MTNR1A | -14.1 | NM_005958 |
| SDC1 | 205.4 | NM_002997 |  | UGT2A1 | -13.0 | NM_006798 |
| HMMR | 200.6 | NM_012485 |  | B7H3 | -13.0 | NM_025240 |
| CDC45L | 186.2 | NM_003504 |  | LOC378204 | -13.0 | XM_353706 |
| POLQ | 181.0 | NM_006596 |  | ARSD | -12.9 | NM_009589 |
| CDCA2 | 172.5 | NM_152562 |  | FLJ11539 | -12.2 | NM_024748 |
| KIF11 | 168.3 | NM_004523 |  | HABP2 | -12.2 | NM_004132 |
| UHRF1 | 167.1 | NM_013282 |  | ITLN1 | -12.2 | NM_017625 |
| MGC19780 | 156.4 | NM_144988 |  | MAP17 | -11.5 | NM_005764 |
| FTHFSDC1 | 128.9 | NM_015440 |  | LOC119692 | -11.1 | XM_061624 |
| GLDC | 118.2 | NM_000170 |  | PTPRE | -10.9 | NM_130435 |
| KIFC1 | 107.4 | XM_352965 |  | SLC6A8 | -10.8 | NM_005629 |
| EAF2 | 104.5 | NM_018456 |  | LOC388007 | -10.8 | XM_370772 |
| TRIP | 98.2 | NM_005879 |  | IGFBP1 | -10.4 | NM_000596 |
| ASPM | 95.5 | NM_018136 |  | GAP43 | -10.4 | NM_002045 |
| CCNA2 | 93.9 | NM_001237 |  | SPTB | -10.3 | NM_000347 |
| MGC45866 | 93.5 | NM_152259 |  | KIAA1217 | -10.0 | NM_019590 |
| CENPA | 92.7 | NM_001809 |  | C20orf54 | -9.9 | NM_033409 |
| TRIP13 | 90.2 | NM_004237 |  | TNS | -9.7 | NM_022648 |
| CDCA3 | 89.7 | NM_031299 |  | ARTN | -9.7 | NM_057160 |
| APOBEC3B | 84.5 | NM_004900 |  | SNCA | -9.5 | NM_007308 |
| STAP2 | 81.3 | NM_017720 |  | LOC142910 | -9.4 | XM_084377 |
| SPATS2 | 78.3 | NM_023071 |  | MXI1 | -9.4 | NM_005962 |
| MCM10 | 77.4 | NM_018518 |  | BCL2L1 | -9.3 | NM_138578 |
| DHFR | 68.8 | NM_000791 |  | GLS2 | -9.1 | NM_138566 |
| KNTC2 | 68.1 | NM_006101 |  | OSBP2 | -9.1 | NM_030758 |
| HKE2 | 67.5 | NM_014260 |  | ALS2CR2 | -9.0 | NM_018571 |
| COQ3 | 66.2 | NM_017421 |  | KRT1 | -8.9 | NM_006121 |
| AQP3 | 66.1 | NM_004925 |  | STARD6 | -8.9 | NM_139171 |
| GPRC5D | 65.2 | NM_018654 |  | LOC377522 | -8.8 | XM_352658 |
| FLJ20641 | 64.8 | NM_017915 |  | LOC374730 | -8.7 | XM_351070 |
| OIP5 | 64.7 | NM_007280 |  | FLJ32009 | -8.6 | NM_152718 |
| CSE1L | 64.5 | NM_001316 |  | CA9 | -8.6 | NM_001216 |
| CTPS | 63.4 | NM_001905 |  | RAC3 | -8.5 | NM_005052 |
| BUB1B | 61.8 | NM_001211 |  | FLJ25006 | -8.4 | NM_144610 |
| IGSF4 | 61.5 | NM_014333 |  | PROS1 | -8.3 | NM_000313 |
| UCHL1 | 57.1 | NM_004181 |  | GMPPB | -8.2 | NM_021971 |
| RAB30 | 55.4 | NM_014488 |  | CTXL | -8.1 | NM_014312 |
| TOPK | 54.5 | NM_018492 |  | ADAM28 | -8.1 | NM_021777 |
| SLC25A10 | 54.4 | NM_012140 |  | LOC134147 | -8.0 | NM_138809 |
| MELK | 53.1 | NM_014791 |  | MYBPH | -7.8 | NM_004997 |
| SGCB | 52.8 | NM_000232 |  | LOC360200 | -7.8 | NM_182973 |
| KIF23 | 50.1 | NM_004856 |  | NOV | -7.7 | X96584 |
| MRPL40 | 48.6 | NM_003776 |  | LOC374843 | -7.6 | XM_351156 |
| GGH | 48.0 | NM_003878 |  | MGC26484 | -7.5 | XM_374608 |
| PDHX | 47.9 | NM_003477 |  | RAP1GA1 | -7.4 | NM_002885 |
| DNAJC3 | 46.8 | NM_006260 |  | LOC390611 | -7.3 | XM_372581 |
| ELL2 | 46.8 | NM_012081 |  | MGC32871 | -7.3 | NM_152311 |
| FOXM1 | 45.7 | NM_021953 |  | PHEX | -7.3 | NM_000444 |
| CDC2 | 45.4 | NM_033379 |  | FLJ25124 | -7.2 | NM_144698 |
| SMC2L1 | 45.1 | NM_006444 |  | LOC139431 | -7.2 | XM_066695 |
| STK6 | 44.6 | NM_198437 |  | LOC401169 | -7.2 | XM_379306 |
| HCAP-G | 44.3 | NM_022346 |  | CHRM5 | -7.2 | NM_012125 |
| C14orf145 | 43.4 | NM_152446 |  | FLJ20701 | -6.9 | NM_017933 |
| C7orf24 | 43.3 | NM_024051 |  | GPR146 | -6.8 | NM_138445 |
| CENPF | 43.2 | NM_016343 |  | PLEK2 | -6.7 | NM_016445 |
| COX7A3 | 42.9 | NM_183003 |  | ALAS2 | -6.7 | NM_000032 |
| RPC8 | 42.4 | NM_138338 |  | CA1 | -6.6 | NM_001738 |
| COBLL1 | 41.7 | NM_014900 |  | SOX12 | -6.6 | NM_006943 |
| HRASLS2 | 41.0 | NM_017878 |  | PTGFRN | -6.5 | XM_040709 |
| LOC92755 | 40.9 | XM_047083 |  | LOC402429 | -6.5 | XM_378143 |
| LOC376333 | 40.0 | XM_352172 |  | NFIX | -6.5 | NM_002501 |
| LOC400869 | 40.0 | XM_379014 |  | FCER1A | -6.5 | NM_002001 |
| RPL34 | 38.7 | NM_000995 |  | IGSF3 | -6.4 | NM_001542 |
| MGC5528 | 37.7 | NM_024094 |  | TRIM10 | -6.4 | NM_006778 |
| SMC4L1 | 36.6 | NM_005496 |  | LOC388199 | -6.4 | XM_370924 |
| SARA2 | 36.4 | NM_016103 |  | SLC36A3 | -6.4 | NM_181774 |
| CDCA2 | 36.3 | NM_152562 |  | LOC219870 | -6.4 | XM_166777 |
| CCNB1 | 35.5 | NM_031966 |  | GYPE | -6.4 | NM_002102 |
| LOC377524 | 34.5 | XM_352660 |  | LOC219952 | -6.4 | XM_166910 |
| MT1K | 34.2 | NM_176870 |  | F2RL1 | -6.3 | NM_005242 |
| SLC25A4 | 33.7 | NM_001151 |  | CAMK2A | -6.2 | NM_171825 |
| TAF9 | 32.8 | NM_016283 |  | HBG1 | -6.2 | NM_000559 |
| KIF4A | 32.8 | NM_012310 |  | SFRP2 | -6.2 | XM_050625 |
| PHAX | 32.1 | NM_032177 |  | KIAA1892 | -6.2 | NM_015397 |
| JUP | 32.1 | NM_002230 |  | HBG2 | -6.2 | NM_000184 |
| ITGB3BP | 31.9 | NM_014288 |  | LOC375765 | -6.1 | XM_353490 |
| WEE1 | 31.7 | NM_003390 |  | BCR | -6.1 | NM_021574 |
| LNPEP | 31.2 | NM_005575 |  | TM4SF9 | -6.1 | NM_005723 |
| MTB | 30.9 | NM_017760 |  | KRT23 | -6.1 | NM_173213 |
| TZFP | 30.4 | NM_014383 |  | PRO2015 | -6.1 | NM_018512 |
| MGC17301 | 30.3 | NM_152637 |  | CACNG6 | -6.1 | NM_145814 |
| MGC33424 | 29.5 | NM_153705 |  | ST5 | -6.1 | S45936 |
| BF | 29.4 | NM_001710 |  | ALPL | -6.0 | NM_000478 |
| KNSL7 | 28.5 | NM_020242 |  | LOC400768 | -6.0 | XM_378883 |
| RAMP | 27.8 | NM_016448 |  | RPIP8 | -6.0 | NM_006695 |
| Pfs2 | 27.8 | NM_016095 |  | LOC389450 | -6.0 | XM_374194 |
| DHX9 | 27.5 | NM_001357 |  | MTND6 | -6.0 | NM_173714 |
| UBE2C | 27.4 | NM_181802 |  | GLI2 | -6.0 | NM_005270 |
| GADD45G | 27.2 | NM_006705 |  | TRIM10 | -6.0 | NM_006778 |
| RASGRP3 | 26.1 | NM_170672 |  | LOC283159 | -5.9 | XM_208543 |
|  |  |  |  |  |  |  |
