# Supplementary material for: Characterization of early host responses in adults with dengue disease
Source: BMC Infect Dis. 2011 Aug 2;11:209. doi: 10.1186/1471-2334-11-209 (PMC3163546; doi:10.1186/1471-2334-11-209)
Supplement: Additional file 3 — The top 100 differentially abundant transcripts in samples from patients with acute dengue relative to samples from febrile patients with non-dengue disease. A table outlining the top 100 differentially abundant transcripts in samples from patients with acute dengue relative to samples from febrile patients with non-dengue disease. [file 1471-2334-11-209-S3.DOC]

Additional file 3. The top 100 differentially abundant transcripts in samples from patients with acute dengue relative to samples from febrile patients with non-dengue disease.

| **Symbol** | **Fold change** | **NCBI accession** |  | **Symbol** | **Fold change** | **NCBI accession** |
| --- | --- | --- | --- | --- | --- | --- |
| CCL2 | 438.2 | NM_002982 |  | C4BPA | -12.1 | NM_000715 |
| JUP | 54.7 | NM_002230 |  | PI3 | -11.2 | NM_002638 |
| DEFB1 | 42.9 | NM_005218 |  | TMG4 | -8.3 | NM_024081 |
| NMES1 | 38.2 | NM_032413 |  | KRTAP11-1 | -7.4 | NM_175858 |
| CCNA1 | 34.6 | NM_003914 |  | TGFA | -6.7 | NM_003236 |
| RNASE1 | 27.0 | NM_198232 |  | GAP43 | -6.5 | NM_002045 |
| TGM1 | 24.2 | NM_000359 |  | SPAP1 | -6.4 | NM_138739 |
| LOC375110 | 21.9 | XM_353248 |  | CYP27A1 | -5.8 | NM_000784 |
| CCL8 | 21.6 | NM_005623 |  | TM6SF1 | -5.6 | NM_023003 |
| MT1K | 21.5 | NM_176870 |  | F2RL1 | -5.5 | NM_005242 |
| IFI27 | 18.6 | NM_005532 |  | VENTX2 | -5.5 | NM_014468 |
| USP18 | 18.4 | NM_017414 |  | KIAA0794 | -5.1 | XM_087353 |
| LOC377812 | 16.8 | XM_352798 |  | VNN2 | -5.0 | NM_004665 |
| TRIM14 | 16.0 | NM_014788 |  | MGC18216 | -5.0 | NM_152452 |
| ARP10 | 15.7 | NM_181773 |  | SLC2A14 | -4.9 | NM_153449 |
| PKD2L1 | 15.2 | NM_016112 |  | LOC378204 | -4.7 | XM_353706 |
| AXL | 12.5 | M76125 |  | CA4 | -4.6 | NM_000717 |
| BF | 11.7 | NM_001710 |  | BLR1 | -4.6 | NM_032966 |
| LGMN | 11.3 | NM_005606 |  | UGT2A1 | -4.4 | NM_006798 |
| OTOF | 11.0 | NM_194322 |  | LOC360200 | -4.3 | NM_182973 |
| RGL1 | 9.7 | NM_015149 |  | MME | -4.3 | NM_000902 |
| STI2 | 9.5 | NM_145755 |  | ALPL | -4.3 | NM_000478 |
| IL4I1 | 8.4 | NM_172374 |  | POLR2B | -4.3 | NM_000938 |
| Hes4 | 8.2 | NM_021170 |  | C14orf78 | -4.2 | XM_290629 |
| SYTL4 | 7.9 | NM_080737 |  | ING5 | -4.2 | NM_032329 |
| PLA2G4C | 7.9 | NM_003706 |  | KRT23 | -4.1 | NM_173213 |
| SIGLEC11 | 7.7 | NM_052884 |  | KIAA0701 | -4.1 | XM_045423 |
| NR1H3 | 6.5 | NM_005693 |  | C20orf36 | -4.0 | NM_018257 |
| RASGRP3 | 6.5 | NM_170672 |  | ZNF537 | -4.0 | NM_020856 |
| HINT3 | 6.4 | NM_138571 |  | CCT6A | -4.0 | NM_001762 |
| TCN2 | 6.2 | NM_000355 |  | ATP1B1 | -3.9 | NM_001677 |
| LAMP3 | 6.2 | NM_014398 |  | RALB | -3.9 | M35416 |
| EPHB2 | 6.1 | NM_004442 |  | ZFP36L2 | -3.9 | NM_006887 |
| AKAP2 | 6.1 | NM_007203 |  | VPREB3 | -3.9 | NM_013378 |
| MDK | 6.1 | NM_002391 |  | CPD | -3.9 | NM_001304 |
| LOC400759 | 6.0 | XM_375746 |  | SCA1 | -3.8 | NM_000332 |
| LGALS3BP | 5.8 | NM_005567 |  | LOC284064 | -3.8 | XM_210334 |
| HIRIP3 | 5.8 | NM_003609 |  | FLJ40584 | -3.8 | XM_069189 |
| MJD | 5.8 | NM_004993 |  | AVIL | -3.8 | NM_006576 |
| HESX1 | 5.7 | NM_003865 |  | SULT1B1 | -3.8 | NM_014465 |
| CXCL10 | 5.7 | NM_001565 |  | FLJ34218 | -3.7 | NM_152470 |
| C1QG | 5.6 | NM_172369 |  | CAMK1D | -3.7 | NM_153498 |
| DNAPTP6 | 5.6 | NM_015535 |  | LOC387760 | -3.7 | XM_373494 |
| SAMD4 | 5.6 | NM_015589 |  | TNFSF5 | -3.7 | NM_000074 |
| CMKLR1 | 5.6 | NM_004072 |  | TRPM6 | -3.7 | NM_017662 |
| RPEL1 | 5.6 | XM_166420 |  | EMR3 | -3.7 | NM_152939 |
| FOXC1 | 5.5 | NM_001453 |  | FLJ14166 | -3.6 | NM_024565 |
| ABTB2 | 5.4 | NM_145804 |  | ZDHHC19 | -3.6 | NM_144637 |
| NRIP1 | 5.3 | NM_003489 |  | NELL2 | -3.6 | NM_006159 |
| PDGFRB | 5.2 | NM_002609 |  | FLJ25416 | -3.6 | NM_145018 |
| RIN2 | 5.1 | NM_018993 |  | SELENBP1 | -3.6 | NM_003944 |
| BRDG1 | 5.1 | NM_012108 |  | GAMT | -3.6 | NM_138924 |
| TRIM6 | 4.9 | NM_058166 |  | BCL2L1 | -3.6 | NM_138578 |
| COL8A2 | 4.8 | NM_005202 |  | C9orf45 | -3.6 | NM_030814 |
| P2RY6 | 4.8 | NM_176798 |  | SPAP1 | -3.5 | NM_138739 |
| PI4K2B | 4.8 | NM_018323 |  | LOC341315 | -3.4 | XM_292012 |
| MT1J | 4.7 | NM_175622 |  | ELYS | -3.4 | NM_175865 |
| CTSL | 4.7 | NM_001912 |  | LIMS2 | -3.4 | NM_017980 |
| RIN1 | 4.6 | NM_004292 |  | LOC129531 | -3.4 | NM_138798 |
| LY6E | 4.5 | NM_002346 |  | PPM1A | -3.3 | NM_177951 |
| MT2A | 4.4 | NM_005953 |  | ZNF541 | -3.3 | NM_032255 |
| G1P2 | 4.4 | NM_005101 |  | C9orf87 | -3.3 | NM_018112 |
| KIF23 | 4.3 | NM_004856 |  | HSF2 | -3.3 | NM_004506 |
| MGC15416 | 4.3 | NM_032371 |  | PIP5K1A | -3.3 | NM_003557 |
| KIAA1618 | 4.3 | NM_020954 |  | FLJ12895 | -3.3 | NM_023926 |
| LAG3 | 4.3 | NM_002286 |  | ANXA3 | -3.2 | NM_005139 |
| FN3K | 4.2 | NM_022158 |  | ZNF238 | -3.2 | NM_205768 |
| H1F0 | 4.1 | NM_005318 |  | LOC283663 | -3.2 | XM_378514 |
| OSBPL5 | 4.1 | NM_145638 |  | LOC389185 | -3.2 | XM_374067 |
| KIAA1237 | 4.0 | XM_087386 |  | LOC138881 | -3.2 | XM_071150 |
| TRIM5 | 4.0 | NM_033092 |  | CACNG6 | -3.1 | NM_145814 |
| LGP2 | 4.0 | NM_024119 |  | RASGRP1 | -3.1 | NM_005739 |
| ABCD1 | 3.9 | NM_000033 |  | D2LIC | -3.1 | NM_015522 |
| SRCRB4D | 3.9 | NM_080744 |  | MGC46336 | -3.1 | XM_290712 |
| FLJ37045 | 3.8 | NM_175889 |  | FLJ32955 | -3.1 | NM_153041 |
| cig5 | 3.8 | NM_080657 |  | MICAL2 | -3.1 | NM_014632 |
| OASL | 3.8 | NM_198213 |  | SESN3 | -3.1 | NM_144665 |
| AKAP2 | 3.7 | NM_007203 |  | HIP14 | -3.1 | NM_015336 |
| PNPT1 | 3.7 | NM_033109 |  | BTG4 | -3.1 | NM_017589 |
| TFEC | 3.7 | NM_012252 |  | CDR2 | -3.0 | XM_071866 |
| FLJ10815 | 3.6 | NM_018231 |  | ACCN1 | -3.0 | NM_183377 |
| C1QB | 3.6 | NM_000491 |  | LCHN | -3.0 | XM_379938 |
| C1orf29 | 3.6 | NM_006820 |  | KIAA0133 | -3.0 | NM_014777 |
| ZNF145 | 3.5 | NM_006006 |  | EIF4ENIF1 | -3.0 | NM_019843 |
| OAS2 | 3.5 | NM_016817 |  | C13orf10 | -3.0 | NM_022118 |
| CD38 | 3.5 | NM_001775 |  | LOC168850 | -3.0 | NM_176814 |
| KCTD14 | 3.5 | NM_023930 |  | LOC91942 | -3.0 | NM_174889 |
| ACP2 | 3.5 | NM_001610 |  | HNRPH2 | -3.0 | NM_019597 |
| FDXR | 3.4 | NM_024417 |  | SLPI | -3.0 | NM_003064 |
| SLC3A2 | 3.4 | NM_002394 |  | CACNG1 | -3.0 | NM_000727 |
| CD209 | 3.4 | NM_021155 |  | ELOVL5 | -2.9 | NM_021814 |
| OAS3 | 3.4 | NM_006187 |  | MS4A1 | -2.9 | NM_021950 |
| DDB2 | 3.3 | NM_000107 |  | LOC375765 | -2.9 | XM_353490 |
| CCL1 | 3.3 | NM_002981 |  | MYF6 | -2.9 | NM_002469 |
| TRIM14 | 3.3 | NM_014788 |  | RAB22A | -2.9 | NM_020673 |
| LOC254359 | 3.3 | XM_170667 |  | MARLIN1 | -2.9 | NM_144720 |
| IFIT1 | 3.3 | NM_001548 |  | FLJ11336 | -2.9 | NM_018393 |
| NEXN | 3.3 | NM_144573 |  | PROS1 | -2.9 | NM_000313 |
| GNGT2 | 3.3 | NM_031498 |  | NEU2 | -2.9 | NM_005383 |
| C9orf91 | 3.2 | NM_153045 |  | KIAA1265 | -2.8 | XM_047707 |
