# Supplementary material for: Characterization of early host responses in adults with dengue disease
Source: BMC Infect Dis. 2011 Aug 2;11:209. doi: 10.1186/1471-2334-11-209 (PMC3163546; doi:10.1186/1471-2334-11-209)
Supplement: Additional file 4 — Differentially abundant transcripts in samples taken at defervescence from DENV RT-PCR/DENV-IgG positive patients at inclusion relative to DENV RT-PCR positive/DENV-IgG negative patients at inclusion. A table outlining the differentially abundant transcripts in samples taken at defervescence from DENV RT-PCR/DENV-IgG positive patients at inclusion relative to DENV RT-PCR positive/DENV-IgG negative patients at inclusion. [file 1471-2334-11-209-S4.DOC]

Additional file 4. Differentially abundant transcripts in samples taken at defervescence from DENV RT-PCR / DENV-IgG positive patients at inclusion relative to DENV RT-PCR positive / DENV-IgG negative patients at inclusion.

| **Symbol** | **Fold change** | **NCBI accession** |
| --- | --- | --- |
| CCR2 | 16.1 | NM_000648 |
| KCTD3 | 10.5 | NM_016121 |
| CHORDC1 | 7.8 | NM_012124 |
| SNX16 | 5.8 | NM_152837 |
