# Supplementary material for: Characterization of early host responses in adults with dengue disease
Source: BMC Infect Dis. 2011 Aug 2;11:209. doi: 10.1186/1471-2334-11-209 (PMC3163546; doi:10.1186/1471-2334-11-209)
Supplement: Additional file 5 — Differentially abundant transcripts in samples taken at convalescence from DENV RT-PCR/DENV-IgG positive patients at inclusion relative to DENV RT-PCR positive/DENV-IgG negative patients at inclusion. A table outlining the differentially abundant transcripts in samples taken at convalescence from DENV RT-PCR/DENV-IgG positive patients at inclusion relative to DENV RT-PCR positive/DENV-IgG negative patients at inclusion. [file 1471-2334-11-209-S5.DOC]

Additional file 5. Differentially abundant transcripts in samples taken at convalescence from DENV RT-PCR / DENV-IgG positive patients at inclusion relative to DENV RT-PCR positive / DENV-IgG negative patients at inclusion.

| **Symbol** | **Fold change** | **NCBI accession** |  | **Symbol** | **Fold change** | **NCBI accession** |
| --- | --- | --- | --- | --- | --- | --- |
| IQGAP3 | 7.5 | NM_178229 |  | TUBB | -4.9 | NM_001069 |
| OLIG2 | 6.0 | NM_005806 |  | EFNB2 | -4.4 | NM_004093 |
| KIAA0794 | 5.8 | XM_087353 |  | LOC376138 | -3.4 | XM_352084 |
| KIAA1354 | 5.4 | NM_018847 |  | ORM1 | -2.9 | NM_000607 |
| GAJ | 5.1 | NM_032117 |  | DKFZp434C0631 | -2.8 | NM_173498 |
| CTSL | 4.7 | NM_145918 |  | LOC374370 | -2.6 | XM_353000 |
| ARF4L | 4.6 | NM_001661 |  | BGALT15 | -2.6 | NM_198540 |
| LOC376939 | 4.6 | XM_352472 |  | CD200R | -2.5 | NM_138940 |
| DRB1 | 4.3 | NM_152945 |  | PXMP4 | -2.3 | NM_183397 |
| CBFA2T2 | 4.2 | NM_005093 |  | LOC377653 | -2.2 | XM_352732 |
| KUB3 | 3.7 | NM_033276 |  | PAPPA | -2.1 | NM_002581 |
| LOC89894 | 3.6 | NM_138341 |  | LTF | -2.1 | NM_002343 |
| ZNF193 | 3.5 | NM_006299 |  | RANBP2 | -2.0 | NM_006267 |
| FLJ22729 | 3.5 | NM_024683 |  |  |  |  |
| MGC20262 | 3.5 | NM_152421 |  |  |  |  |
| KIAA1618 | 3.3 | NM_020954 |  |  |  |  |
| CCL3L1 | 3.3 | NM_021006 |  |  |  |  |
| TOR1B | 3.2 | NM_014506 |  |  |  |  |
